# Acute Non-Suppurative Encephalitis

**Published:** 1903-09-19

**Authors:** 


### ACUTE NON-SUPPURATIVE ENCEPHALITIS.

IN dealing with this condition, Dr. H. Brooks[1] considers it probable that the disease is much more frequent than is generally supposed, and that its reported rarity is partly due to the great difficulty of differential diagnosis. The symptoms are so similar to those of many other conditions that doubtless clinical cases are often misinterpreted. Furthermore, the gross lesions may be so slight as to throw no additional light on the case, even after post-mortem investigation. Microscopical examination, in a considerable number of instances, is the only certain method of detecting the lesion. It is generally admitted that a considerable number of cases recover, either wholly or in part, according to the extent and location of the encephalitis. Dr. Brooks points out that the restricted notions that are in vogue of its etiology may account to a great extent for the failures in diagnosis, and asks why every agent which is able to set up inflammatory processes in other parts of the body, as in the liver and kidneys, should not be accepted as an etiological factor in encephalitis. Considering the brain in the same broad pathological sense as other more fully understood organs, it would be quite consistent to include nearly all exciters of inflammation as possible etiological factors in encephalitis. Of such agents alcohol is doubtless the most frequent, while metabolic poisons and bacterial toxins must be included. There is no reason why all these factors should not enter into the production of inflammation of the brain, as they do in inflammatory processes in other parts of the body. In addition may be added the inflammation following trauma. Acute encephalitis may terminate in death if the disease involves part of the encephalon, the perfect function of which is essential to life, *e.g.*, the medulla.

[1] Medical News, August 8.

